# Sub-Wavelength Resonances in Metamaterial-Based Multi-Cylinder Configurations

**DOI:** 10.3390/ma4010117

**Published:** 2010-12-31

**Authors:** Samel Arslanagić, Olav Breinbjerg

**Affiliations:** Department of Electrical Engineering, Electromagnetic Systems, Technical University of Denmark, Ørsteds Plads, Bld. 348, DK-2800, Kgs. Lyngby, Denmark; E-Mail: ob@elektro.dtu.dk

**Keywords:** metamaterials, sub-wavelength resonances, scattering, line source

## Abstract

Sub-wavelength resonances known to exist in isolated metamaterial-based structures of circular cylindrical shape are investigated with the purpose of determining whether the individual resonances are retained when several of such resonant structures are grouped to form a new structure. To this end, structures consisting of 1, 2 and 4 sets of metamaterial-based concentric cylinders excited by an electric line current are analyzed numerically. It is demonstrated that these structures recover the resonances of the individual structures even when the cylinders are closely spaced and the new structure is thus electrically small. The investigation is conducted through a detailed analysis of the electric near-field distribution as well as the radiation resistance in those cases where the individual structures are made of simple dielectric materials in conjunction with simple, but lossy and dispersive, metamaterials.

## 1. Introduction

The field of metamaterials (MTMs) has experienced significant scientific advances in recent years, and numerous applications within the microwave [1,2,3] and the optical [4] frequency regions have been devised. Important examples of MTMs include double-negative (DNG) materials, which possess a negative real part of the permittivity and permeability, as well as epsilon-negative (ENG) and mu-negative (MNG) materials, which possess a negative real part of the permittivity and permeability, respectively. Among the numerous reported applications of these MTMs, specific attention has been devoted to their potential of providing sub-wavelength resonant structures of various canonical shapes [5,6,7,8,9,10,11,12,13,14] either when used alone or in combination with double-positive (DPS) materials, which possess a positive real part of permittivity and permeability. In particular, it was shown in [9] that an isolated set of concentric circular MTM-based cylinders excited by a nearby electric line current (ELC) possesses sub-wavelength resonances where the excitation of specific modes is found to lead to large radiated power for constant ELC.

The purpose of the present work is to investigate how the sub-wavelength resonances of the isolated MTM-based concentric cylinder structures studied in [9] are affected when several of such structures are grouped to form a new structure. To this end, configurations consisting of 1, 2 and 4 sets of MTM-based concentric cylinders, henceforth referred to as 1-, 2-, and 4-cylinder structure, are analyzed. It is shown that these structures recover the resonances of the individual structures even when the cylinders are closely spaced and the configuration is thus electrically small. The analysis is conducted with the ANSOFT High Frequency Structural Simulator (HFSS) [15] and includes detailed investigations of the electric near-field distribution and the radiation resistance in case of simple, but lossy and dispersive, MTMs. A collection of MTM-based objects were studied in [6] with the purpose of devising an effective hybrid MTM, in [16] for cloaking purposes, and in [17] for its scattering properties. The present work is an extension of [18], and in comparison, includes both a full account of the HFSS model as well as additional near-field investigations.

The present manuscript is organized as follows. In Section 2, the investigated structures are defined and the analysis techniques, including the exact method used for the 1-cylinder structure, as well as the numerical method, are described. This section also includes a brief discussion on the conditions for sub-wavelength resonance in the isolated 1-cylinder structures; this is used in conjunction with the exact analytical results to define the electrical and geometrical parameters of a given 1-cylinder structure. In Section 3, the numerical results are presented; in particular, the resonances of the individual structures are studied as the distance between the cylinders is changed. In all cases, the resonant structures are made of simple dielectric materials in conjunction with simple, but lossy and dispersive, MTMs, and the resonant properties of all configurations are analyzed through detailed investigations of their electric field distribution and the radiation resistance. Section 4 includes a summary and conclusion of the present work. The time factor exp(*jωt*, with *ω* being angular frequency and *t* time, is assumed throughout the manuscript.

## 2. Configuration and Theory

### 2.1. Configuration

**Figure 1:**
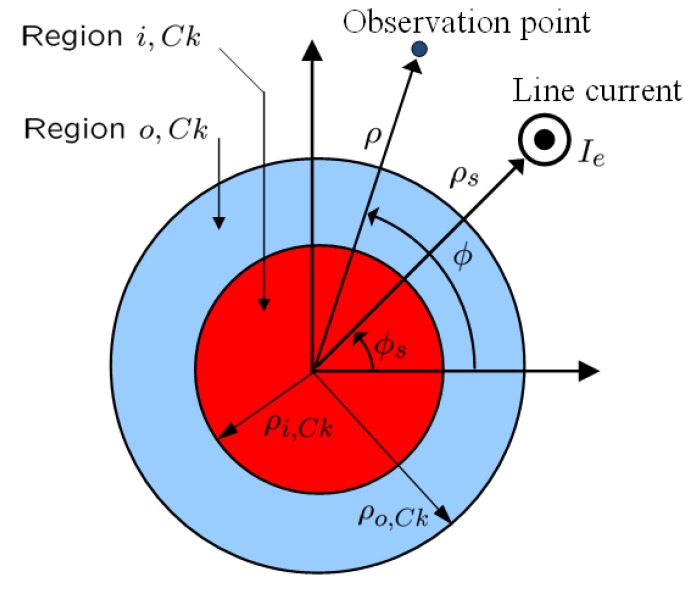
The *k*' th concentric cylinder set of the 1-, 2-, and 4-cylinder structure.

Figure 1 shows the*k*’th concentric cylinder set (*Ck*) of the 1-, 2- or 4-cylinder structures investigated in the present work. A circular cylinder (region *i,Ck*) with center at *O_Ck_* and radius *ρ_i,Ck_* is covered by a concentric circular shell (region *o,Ck*) of outer radius *ρ_o,Ck_*, and located in free space with the permittivity *ε*_0_, permeability *μ*_0_, wave number $k_{0}=\omega \sqrt{\varepsilon_{0}\mu_{0}}>0$ and intrinsic impedance $\eta_{0}=\sqrt{\mu_{0}/\varepsilon_{0}}>0$. Region *i,Ck* (*o,Ck*) consists of simple, and generally lossy and dispersive, DPS, DNG, and/or ENG and MNG materials with the permittivity $\varepsilon_{i,Ck}=\varepsilon_{i,Ck}^{\prime }-j\varepsilon_{i,Ck}^{\prime \prime }\left(\varepsilon_{o,Ek}=\varepsilon_{o,Ek}^{\prime }-j\varepsilon_{o,Ck}^{\prime \prime }\right)$ and the permeability $\mu_{i,Ck}=\mu_{i,Ck}^{\prime }-j\mu_{i,Ck}^{\prime \prime }$ $\left(\mu_{o,Ck}=\mu_{o,Ck}^{\prime }-j\mu_{o,Ck}^{\prime \prime }\right)$ . The 1-, 2-, or 4-cylinder structure is illuminated by an infinite electric line current (ELC) *I_e_* that is parallel to the cylinders and can be located in any of the regions. The cylindrical (*ρ,ϕ,z*)-coordinate system and the Cartesian (*x,y,z*)-coordinate system are introduced with the *z*-axis coinciding with the common axis of the cylinders. The coordinates of the observation point are (*ρ,ϕ*), while those of the ELC are (*ρ_s_,ϕ_s_*).

The 1-cylinder structure consist of a cylinder *C1* which has its center at the origin, *i.e*., *O*_*C*1_ = (0,0). The 2-cylinder structure consists of the previous cylinder *C1* and a cylinder *C2* having its center at $O_{C2}=(\rho_{o,C1}+d+\rho_{0,C2},0)$. Thus, the cylinder *C2* is displaced along the *x*-axis with a separation distance *d* to the cylinder *C1*. The 4-cylinder structure consists of the previous cylinders *C1* and *C2*, and the additional cylinders *C3* and *C4* with their centers at $O_{C3}=(\rho_{o,C1}+d/2,d/2+\rho_{o,C3})$ and *O*_*C*4_ = $\left(\rho_{o,C1}+d/2,-d/2-\rho_{o,C4}\right)$, respectively.

### 2.2. Analysis Methods

For the 1-cylinder structure, both an exact as well as a numerical solution have been obtained. The exact solution is based on the eigenfunction expansion method, see e.g., [19]. Whereas the details of the exact solution can be found in [9], we emphasize below only the main points. The incident field of the ELC, as well as the unknown fields in the three regions, *i.e.*, the scattered field in the region containing the ELC and total field in the remaining regions, are all expanded in terms of cylindrical wave functions. These expansions represent the multipole expansion of the respective fields, and for the unknown fields, they contain a set of unknown expansion coefficients $A_{jn}$, j=1, 2, 3, and 4 (with *j* = 1 for region *i,Ck*, *j* = 2 and 3 for region *o,Ck*, and *j* = 4 for the ambient free-space medium), where *n* is the mode number with *n* = 0 referring to the monopole mode, *n* = 1 to the dipole mode and so on. The unknown expansion coefficients *A_in_* depend on the electrical and geometrical parameters of the structure in Figure 1 as well as on the location of the ELC, and they are readily determined by enforcing the boundary conditions at the interfaces between the three regions; once these coefficients are known, the fields in the different regions have been determined.

**Figure 2:**
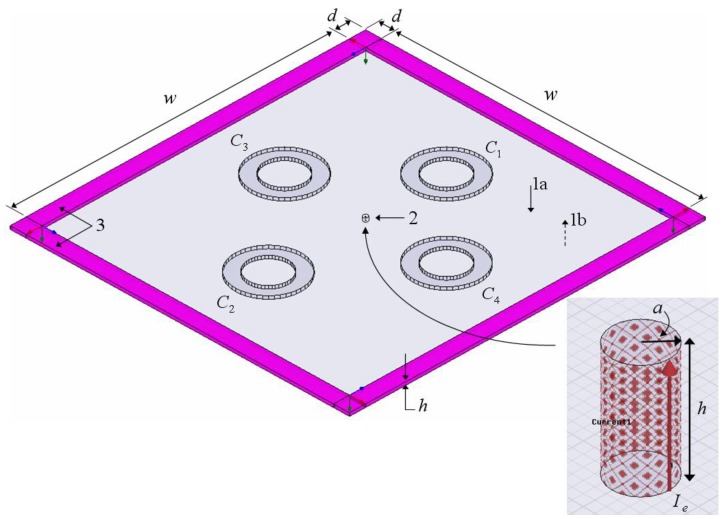
The HFSS model of the 4-cylinder structure. 1: Perfectly conducting top (1a) and bottom (1b) plates; 2: Electric current tube (enlarged in the inset of the figure); 3: Uniform perfect matching layers. The figure is not to scale. See the main text for further explanations.

For the 2- and 4-cylinder structures, a numerical solution is established using the ANSOFT HFSS software [15] (the numerical solution was also employed to investigate the 1-cylinder structure and to compare its results with the exact solution in order to confirm the validity of the established HFSS model). Figure 2 shows the HFSS model, where the 4-cylinder structure with the individual cylinders designated as *C1*, *C2*, *C3*, and *C4* is depicted. The model consists of the ELC source modeled by a finite length current tube of radius *a*, current *I_e_*, and its axis located at ($\rho_{s},\varphi_{s}$) (enlarged in the inset of the figure), and the finite length MTM-based cylinders. The finite length current tube and the MTM-based cylinders are positioned between, and perpendicular to, two parallel, perfectly electrically conducting infinite plates with separation *h.* Due to image theory [19], these plates model the infinite MTM-based cylinders and the ELC. Between the perfectly conducting plates, uniform perfect matching layers which model free-space radiation, which have thickness *d*, circumscribe a square of side length *w*, and have their corners and edges joined, are inserted. The values of the specific parameters of the HFSS model as well additional details are found in Section 3.

### 2.3. Derived Quantities and Resonance Condition

In the present work, the attention is devoted to the radiation resistance, *R_t_*, of the ELC for a given constant value of *I_e_* radiating in the presence of the material structure,

(1) $$R_{t}=\frac{2P_{t}}{I_{e}^{2}}$$

relative to the radiation resistance, *R_i_*, of the ELC with the same current *I_e_* in the absence of the material structure

(2) $$R_{i}=\frac{2P_{i}}{I_{e}^{2}}$$

where the quantities *P_t_* and *P_i_* in the above expressions represent, respectively, the power radiated by the ELC in the presence and absence of the material structure.

For the 1-cylinder structure, the exact expressions for *P_t_* and *P_i_* have been obtained in [9], and are repeated here for the sake of convenience

(3) $$P_{t}=\left(\frac{1}{4}\eta_{0}I_{e}^{2}\right)\left[\frac{k_{0}}{4}\sum_{n=0}^{N_{\max}}\tau_{n}^{2}(3-\tau_{n})|\alpha_{n}{|}^{2}\right]$$

(4) $$P_{i}=\left(\frac{1}{4}\eta_{0}I_{e}^{2}\right)\left[\frac{k_{0}}{2}\right]$$

In (3), the quantity $\alpha_{n}=A_{4n}$ when the ELC is in region *i,Ck* and *o,Ck*, while $\alpha_{n}=J_{n}(k_{0}\rho_{s})+A_{4n}$, where $J_{n}(\cdot )$ is the Bessel function of order *n*, when the ELC is outside the 1-cylinder structure. The symbol $\tau_{n}$ is the Neumann number; thus, $\tau_{n}=1$ for *n* = 0 and $\tau_{n}=2$ otherwise, and *N*_max_ is the truncation limit chosen such to ensure the convergence of the cylindrical wave expansion.

From (3) and (1), it is clear that large values of the total radiation resistance will result if the amplitude of the expansion coefficients *A*_4n_ becomes large. When a given single concentric cylinder set *Ck* is electrically small, *i.e.*, when it is sub-wavelength, these expansion coefficients become very large, and thus exhibit a resonance when the condition is satisfied [6,9]. As explained in [6,9], at least one of the regions comprising such a concentric cylinder set *Ck* must be made of DNG and/or MNG material in order to satisfy the condition in (5), and moreover, the excitation of the sub-wavelength resonances is due to the presence of natural modes in the structure. The resonance condition in (5) has been used in [6,9] to design resonant sub-wavelength 1-cylinder configurations, and is also used next to design the individual concentric cylinder sets of the 1-, 2-, and 4-cylinder structures.

(5) $$\frac{\rho_{i,Ck}}{\rho_{o,Ck}}\approx \sqrt[{2n}]{\frac{\left(\mu_{o,Ck}^{\prime }+\mu_{i,Ck}^{\prime })(\mu_{o,Ck}^{\prime }+\mu_{0}\right)}{\left(\mu_{o,Ck}^{\prime }-\mu_{i,Ck}^{\prime })(\mu_{o,Ck}^{\prime }-\mu_{0}\right)}}$$

## 3. Numerical Results

### 3.1. Resonant Configurations and Further Remarks on the HFSS Model

According to Section 2.3 and [6,9], it is possible to design a sub-wavelength 1-cylinder structure capable of exciting a dipole (*n* = 1) mode resonance, which leads to large values of, e.g., radiated power and radiation resistance. In the present section, we investigate whether these resonances of the individual 1-cylinder structures exist, and under which conditions when several cylindrical structures are grouped to form a new structure. To this end, we choose the individual 1-cylinder configurations which excite the dipole resonances at the design frequencies $f_{0}=\left[250,\;266,\;283,\;300\right]$ MHz for cylinders *C2*, *C3*, *C4* and *C1*, respectively, this leading to the smallest free-space wavelength $\lambda_{0,\min}=1$ m. The excitation of the dipole mode at the specified design frequencies can be accomplished if region i,Ck is free space, *i.e.*, $\left(\varepsilon_{i,Ck},\mu_{i,Ck})=(\varepsilon_{0},\mu_{0}\right)$, region o,Ck is a MNG material with $\left(\varepsilon_{o,Ck},\mu_{o,Ck})=(\varepsilon_{0},-4\mu_{0}\right)$, and $\rho_{i,Ck}=6$ mm, while $\rho_{o,C1}=10.033$ mm, $\rho_{o,C2}=10.024$ mm, $\rho_{o,C3}=10.027$ mm, and $\rho_{o,C4}=10.03$ mm. The values for $\rho_{o,Ck}$ are the exact values of the outer radii of the respective shells of the individual 1-cylinder structures of which the electrical and geometrical parameters are summarized in Table 1.

**Table 1:** Electrical and geometrical parameters of dipolar 1-cylinder configurations.

Cylinder *CK*	*f* [MHz]	*ρ_o,Ck_* [mm]
*C1*	300	10.033
*C2*	250	10.024
*C3*	266	10.027
*C4*	283	10.03
For all structures: $\left(\varepsilon_{i,Ck},\mu_{i,Ck})=(\varepsilon_{0},\mu_{0}\right)$; $\left(\varepsilon_{o,Ck},\mu_{o,Ck})=(\varepsilon_{0},-4\mu_{0}\right)$; $\rho_{i,Ck}=6$ mm		

In all cases, the current of the line source, in the exact as well as the HFSS-based examinations, is set to *I_e_* = 1 A. For the HFSS model, the ELC is modeled by a perfectly conducting tube of radius $a=0.15\;\mathrm{mm}\approx \lambda_{0,\min}/6666$; the side length of the square circumscribed by the uniform perfect matching layers is $w=1000\;\mathrm{mm}=\lambda_{0,\min}$; the separation between the perfectly conducting plates is $h=1\;\mathrm{mm}=\lambda_{0,\min}/1000$, and the thickness of the perfectly matching layers is set to $d=15.81\;\mathrm{mm}\approx \lambda_{0,\min}/63$ (the latter being the default value suggested by HFSS for the chosen value of *w*). The model of the ELC was tested thoroughly by comparing the numerically calculated radiation resistance in free space with the known analytical result. With the current along the tube being equal to *I_e_* = 1 A, the radiation resistance as calculated by HFSS was found to be 0.57 Ω/mm; a value which is very close to the exact analytical result of 0.59 Ω/mm[12], thereby verifying the established HFSS model of the ELC.

In order to assess the frequency behavior of the MNG material of the 1-, 2-, and 4-cylinder structures, the Drude dispersion model [3] has been employed for the permeability $\mu_{o,Ck}$ of region o,Ck for all configurations. In (6), the quantity $\omega_{pm,Ck}$ is the magnetic plasma frequency, and is chosen such that $\mu_{o,Ck}=-4\mu_{0}$ is obtained at the respective design frequencies. The parameter $\gamma_{m,Ck}$ is the magnetic collision frequency representing losses in the material.

(6) $$\mu_{o,Ck}=\mu_{0}\left(1-\frac{\omega_{pm,Ck}^{2}}{\omega (\omega -j\gamma_{m,Ck})}\right)$$

### 3.2. 1-Cylinder Structure

**Figure 3:**
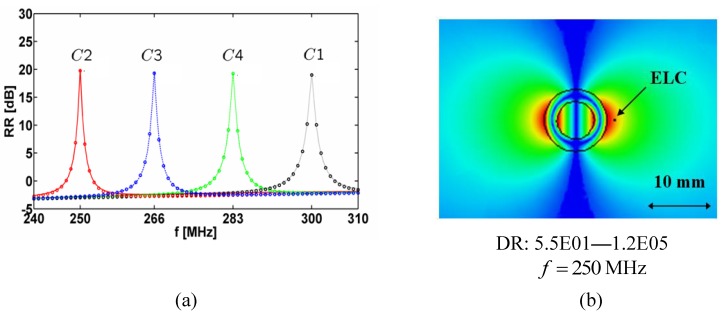
The quantity $\mathrm{RR}=10\log_{10}|R_{t}|$ [dB] as a function of frequency for the resonant 1-cylinder structures (**a**), and the magnitude of the electric field of the resonant *C*2 (250 MHz) 1-cylinder structure for *f* = 250 MHz (**b**). In all cases, each of the individual cylinders are centered at the origin and the ELC is located at $\left(\rho_{s},\varphi_{s})=(\rho_{o,Ck}+2.5\;\mathrm{mm},0{}^{\circ}\right)$. The electric field in (**b**) is depicted in the -plane and the linear dynamic range (DR) in V/m is indicated below the figure. Curves representing the circular surfaces of the structure and a left-right arrow, indicating the size scale of the figure, are likewise indicated in (b).

The resonances of the 1-cylinder structures are illustrated in Figure 3(a) where the quantity $\mathrm{RR}=10\log_{10}|R_{t}|$ [dB], where the radiation resistance *R_t_* (1) has been normalized by 1 Ω/mm, is shown as a function of frequency when each of the cylinders is centered at the origin and the ELC is located at $\left(\rho_{s},\varphi_{s})=(\rho_{o,Ck}+2.5\;\mathrm{mm},0{}^{\circ}\right)$. The permeability of the MNG shell of all configurations is modeled by the lossless Drude model for which $\gamma_{m,Ck}=0$ in all structures. The circles in Figure 3(a) represent the exact analytical results while the full lines represent the corresponding HFSS results. The agreement between the exact analytical results and HFSS results is seen to be excellent; a similar agreement was reported in [13]. It is clear that the individual 1-cylinder structures resonate at the desired designed frequencies; moreover, the values of RR are comparable in the four cases and equal to approximately 20 dB, this showing large enhancements of the radiation resistance of the ELC nearby the MTM-based structures relative to the case where the ELC is alone in free space. Figure 3(b) shows the magnitude of the electric field in the -plane (with the dynamic range (DR) indicated below the figure) of the resonant (MHz) 1-cylinder structure at the frequency *f* =250 MHz (similar electric field results are obtained for other 1-cylinder structures and are therefore not presently included). A clear dipole electric field patter is observed in Figure 3(b), confirming that the resonances in Figure 3(a) are due to the excitation of the dipole mode in the individual cylinders.

It is next investigated how the resonances of the individual 1-cylinder structures reported in Figure 3 are affected when several structures are grouped to form 2- and 4-cylinder structures.

###  3.3. 2-Cylinder Structure

**Figure 4:**
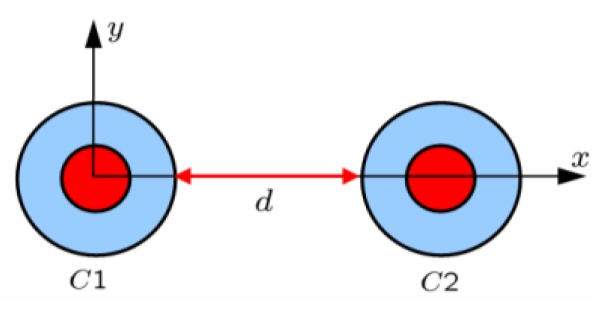

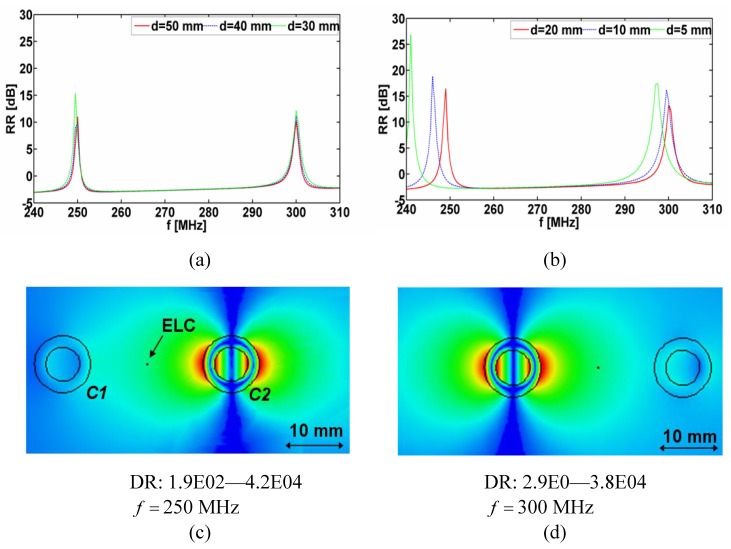
(**a**) and (**b**): The quantity $\mathrm{RR}=10\log_{10}|R_{t}|$ [dB] as a function of frequency for the resonant 2-cylinder structures for different separation distances *d*. The magnitude of the electric field of the resonant 2-cylinder structures for *d* = 40 mm and *f* = 250 MHz; (**c**) and *f* = 300 MHz (**d**). In all cases, the ELC is located at $\left(\rho_{s},\varphi_{s})=(\rho_{o,C1}+d/2,0{}^{\circ}\right)$. The electric field in (c) and (d) is depicted in the *xy*-plane and the linear dynamic range (DR) in V/m is indicated below the figures. Curves representing the circular surfaces of the structure and a left-right arrow, indicating the size scale of the figure, are likewise shown in (c) and (d). Inset above the figure shows the 2-cylinder structure consisting of cylinders *C*1 and *C*2.

**Figure 5:**
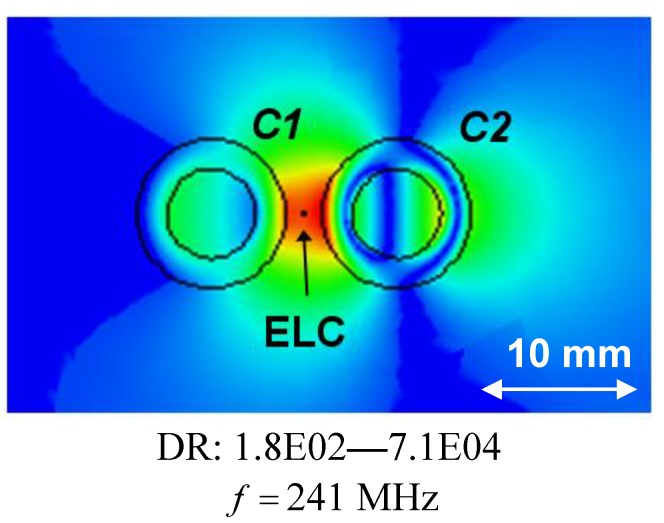
The magnitude of the electric field of the resonant 2-cylinder structure when the separation distance *d* = 5 mm for *f* = 250 MHz, which is the frequency at which the first resonance appears in Figure 4(b). The ELC is located at $\left(\rho_{s},\varphi_{s})=(\rho_{o,C1}+d/2\;\mathrm{mm},0{}^{\circ}\right)$, and the electric field is depicted in the *xy*-plane with the linear dynamic range (DR) in V/m indicated below the figure. Curves representing the circular surfaces of the structure and a left-right arrow, indicating the size scale of the figure, are likewise indicated.

Figure 4 shows the results for the 2-cylinder structure consisting of cylinders *C*1 and *C*2 (the structure is shown in the inset on top of the figure). Specifically, Figure 4(a) and Figure 4(b) show the quantity RR [dB] as a function of frequency for the separation distances *d* = 50, 40, 30, 20, 10 and 5 mm with the ELC located at $(\rho_{s},\phi_{s})=$ $\left(\rho_{i,C1}+d/2,0{}^{\circ}\right)$. For all separation distances *d*, two distinct resonances are found. For *d* = 50 and 40 mm, the resonances occur at *f* = 250 MHz and *f* = 300 MHz, respectively, where the individual cylinders are designed to resonate and their amplitudes are seen to be lower than for the individual cylinders in Figure 3(a), since the ELC is farther away from the cylinders in the present cases. These resonances are due to the dipole mode excitation in the cylinders, as illustrated in Figure 4(c) and (d) where the magnitude of the electric field is shown for *d* = 40 mm and *f* = 250 MHz in (c) and *f* = 300 MHz in (d). As the separation distance *d* decreases further, one and/or both resonances shift slightly away from the resonant frequencies of the isolated individual cylinders. However, these resonances are still due to the dipole modes in the two cylinders. This is, however, not the case for the separation distance of *d* = 5 mm where, e.g., the first resonance at *f* = 241 MHz, which attains higher amplitude than in the case of individual cylinders, is due to a mode characterized by strong coupling between the two cylinders as is illustrated in Figure 5 where the magnitude of the electric field is shown. With the diameter of the individual cylinders being approximately 20 mm, it is thus found that the sub-wavelength resonances of the individual cylinders also occur in 2-cylinder configurations of which the overall size is as small as $\lambda_{0,\min}/20$ (for *d* = 10 mm), thus proving such structures feasible for the potential design of multi-resonant sub-wavelength systems.

### 3.4. 4-Cylinder Structure

**Figure 6:**
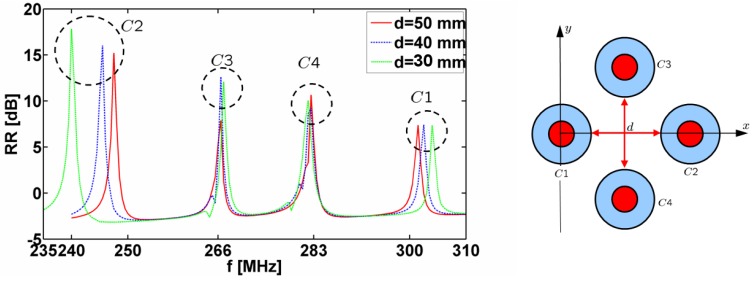
The quantity $\mathrm{RR}=10\log_{10}|R_{t}|$ [dB] as a function of frequency for the resonant 4-cylinder structures for different separation distances *d*. In all cases, the ELC is located at $\left(\rho_{s},\varphi_{s})=(\rho_{o,C1}+d/2,0{}^{\circ}\right)$. The 4-cylinder structure is shown on the right.

Figure 6 shows the results for the 4-cylinder structure (the structure itself is shown in the inset in the right part of the figure).

**Figure 7:**
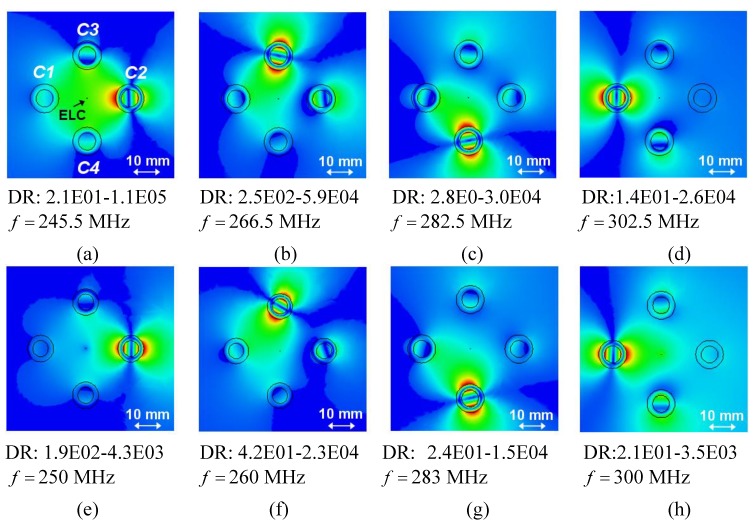
The magnitude of the electric field of the 4-cylinder structure for the separation distance *d* = 40 mm for different frequencies. The ELC is located at $\left(\rho_{s},\varphi_{s})=(\rho_{o,C1}+d/2\;\mathrm{mm},0{}^{\circ}\right)$, and the electric field is depicted in the *xy*-plane with the linear dynamic range (DR) in V/m indicated below the figure. Curves representing the circular surfaces of the structure and a left-right arrow indicating the size scale of the figure, are likewise indicated. (**a**)–(**d**): frequencies at which the resonances occur in Figure 6; (**e**)–(**h**): initial resonance frequencies of the individual structures.

Specifically, Figure 6 shows the quantity $\mathrm{RR}=10\log_{10}|R_{t}|$ [dB] as a function of frequency for the separation distances *d* = 50, 40, and 30 mm when the ELC is located at $\left(\rho_{s},\varphi_{s})=(\rho_{o,C1}+d/2,0{}^{\circ}\right)$. For all separation distances *d*, four distinct resonances are found, although slightly shifted from the resonant frequencies of the individual cylinders and with lower amplitudes than in the case of the 1-cylinder structures in Figure 3(a). For a given separation *d*, this shift is larger for the 4-cylinder than for the 2-cylinder configuration and is seen to be largest for the cylinders *C1* and *C2*. The majority of the resonances in Figure 6 are due to the dipole mode excitation in the individual cylinders; this is clear from Figure 7(a)–(d), which show the magnitude of the electric field (with the dynamic range (DR) indicated below the respective figures) for *d* = 40 mm for the frequencies at which the resonances appear in Figure 6 (*f* = 245.5, 266.5, 282.5, and 302.5 MHz).

**Figure 8:**
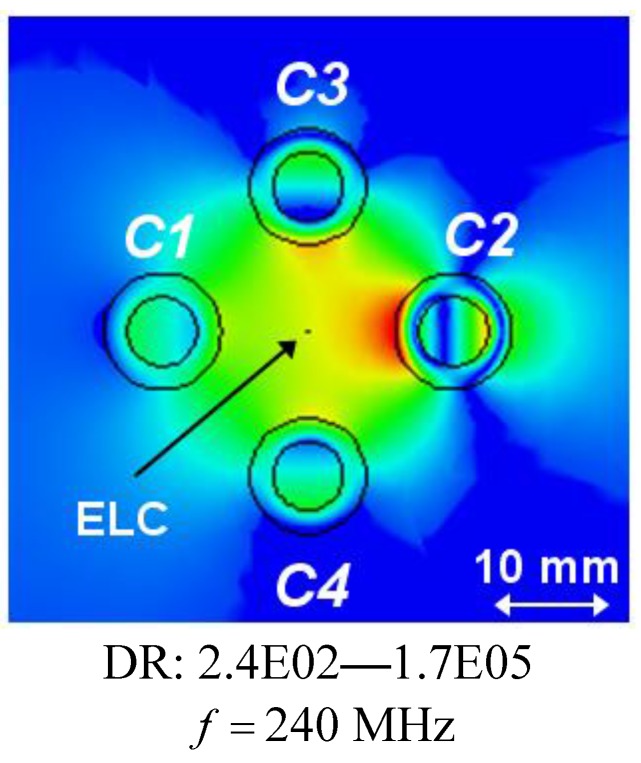
The magnitude of the electric field of the 4-cylinder structure for the separation distance *d* = 30 mm and *f* = 240 MHz; this is the frequency at which the first resonance appears in Figure 6. The ELC is located at $\left(\rho_{s},\varphi_{s})=(\rho_{o,C1}+d/2\;\mathrm{mm},0{}^{\circ}\right)$, and the electric field is depicted in the *xy*-plane with the linear dynamic range (DR) in V/m indicated below the figure. Curves representing the circular surfaces of the structure and a left-right arrow, indicating the size scale of the figure, are likewise indicated.

The corresponding fields at the resonant frequencies of the isolated individual cylinders are shown in Figure 7(e)–(h); these also show clear dipole patterns but with different maximum values of the field. While the field levels for the cylinders *C3* and *C4*, respectively, in Figure 7(b) and (c) are comparable with those in Figure 7(f) and (g), they are at least an order of magnitude lower for cylinders *C2* and *C1*, respectively, in Figure 7(a) and (d), as compared to Figure 7(e) and (h). This explains why, e.g., large RR values are attained for cylinders *C3* and *C4* not only at the frequencies *f* = 266.5 MHz and 282.5 MHz, respectively, but also at the original resonance frequencies of the individual cylinders, whereas this is found not to be the case for cylinders *C2* and *C1*. Moreover, for the separation distance of *d* = 30 mm, the first resonance occurring at *f* = 240 MHz is not due to a clear dipole mode in the cylinder *C*2, but rather to a mode which is due to coupling effects between the four cylinders, as is clearly illustrated by the result in Figure 8, which shows the magnitude of the electric field in this particular case. With the diameter of the individual cylinders being approximately 20 mm, it is thus found that the sub-wavelength resonances of the individual cylinders also occur in 4-cylinder configurations of which the overall size is as small as $\lambda_{0,\min}/12.5$ (for *d* = 40 mm), thus proving such structures feasible for the potential design of multi-resonant sub-wavelength systems.

It is noted that if the individual cylinders are designed such that their resonances are even closer to each other, the coupling becomes more visible than in the case of the presently investigated cylinders. This is supported by the results in Figure 3(a) which suggests that for close enough resonance frequencies, the radiation resistance curves (those parts with significant values of the radiation resistance) for the individual cylinders will considerably overlap each other thus indicating a stronger coupling.

**Figure 9:**
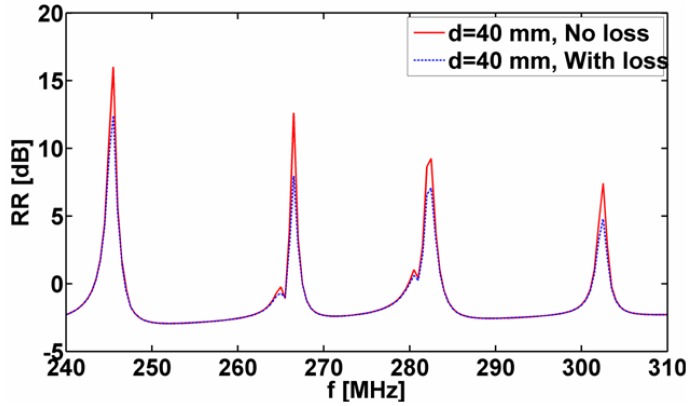
The quantity $\mathrm{RR}=10\log_{10}|R_{t}|$ [dB] as a function of frequency for the lossy and lossless 4-cylinder structures for separation distance *d* = 40 mm. In all cases, the ELC is located at $\left(\rho_{s},\varphi_{s})=(\rho_{o,C1}+d/2,0{}^{\circ}\right)$. See the main text for further explanations.

The analysis thus far has concentrated on the cases where the MNG material of the 1-, 2-. and 4-cylinder structures was modeled by a lossless Drude dispersion model for which $\gamma_{m,Ck}=0$. Presently, the influence of loss on the reported resonant properties of these structures is assessed by incorporating loss in the model. More specifically, the parameter $\gamma_{m,Ck}=0$ was set to $10^{-3}\;2\pi \;f_{0}$, where *f*_0_ is the design frequency of the respective cylinders, and the radiation resistance was for the 4-cylinder configuration with the separation distance *d* = 40 mm. The obtained results are reported in Figure 9 in terms of the quantity $\mathrm{RR}=10\log_{10}|R_{t}|$ [dB] as a function of frequency. This figure also includes the corresponding lossless-case result for comparison purposes. It is observed that resonances occur at the same frequencies as in the lossless case, but that the corresponding amplitudes, as expected, are reduced.

## 4. Summary and Conclusions

This work presented a detailed study of resonant properties of a number of sub-wavelength MTM-based structures of circular cylindrical shape. In particular, attention was devoted to sub-wavelength resonances known to exist in isolated MTM-based structures of circular cylindrical shape with the aim of determining whether the individual resonances are retained when several of such resonant structures are grouped to form a new structure. To this end, structures composed of 1, 2 and 4 sets of MTM-based concentric cylinders excited by an ELC were analyzed numerically in ANSOFT HFSS with regard to their near-field properties and radiation resistance. The MTMs of the individual structures were assumed to be simple, but lossy and dispersive, where the effects of the latter were accounted for by the Drude dispersion mode.

It was demonstrated that the sub-wavelength resonances of the isolated MTM-based concentric cylinder structures also occur for the structures composed of 2 and 4 sets of MTM-based concentric cylinders even in the case where the cylinders are closely spaced and the entire structure is thus electrically small. Specifically, overall sizes of about 1/20 and 1/12.5 of the smallest free-space wavelength were found for 2- and 4-cylinder structures, respectively, in which the respective resonances were due to the dipole mode excitation in the constituent cylinders. These MTM-based structures thus offer the possibility for multi-resonant sub-wavelength configurations.
